# Interactome-transcriptome analysis reveals the high centrality of genes differentially expressed in lung cancer tissues

**DOI:** 10.1093/bioinformatics/bti688

**Published:** 2005-09-27

**Authors:** Shinichiro Wachi, Ken Yoneda, Reen Wu

**Affiliations:** Center for Comparative Respiratory Biology and Medicine and Division of Pulmonary/Critical Care Medicine, University of California, Davis, CA 95616, USA

## Abstract

**Motivation:**

Global protein interaction network (interactome) analysis provides an effective way to understand the relationships between genes. Through this approach, it was demonstrated that the essential genes in yeast tend to be highly connected as well as connected to other highly connected genes. This is in contrast to the genes that are not essential, which share neither of these properties. Using a similar interactome-transcriptome approach, the topological features in the interactome of differentially expressed genes in lung squamous cancer tissues are assessed.

**Results:**

This analysis reveals that the genes that are differentially elevated, as obtained from the microarray gene profiling data, in cancer are well connected, whereas the suppressed genes and randomly selected ones are less so. These results support the notion that a topological analysis of cancer genes using protein interaction data will allow the placement of the list of genes, often of the disparate nature, into the global, systematic context of the cell. The result of this type of analysis may provide the rationale for therapeutic targets in cancer treatment.

## 1 Introduction

To adapt a famous passage of John Donne, no gene is an island. Systems biology, or more specifically network biology, is driven by the gradual realization that a single gene is seldom accountable for a discrete biological function (Barabasi and Oltvai, 2004). In response, contemporary biology has amassed a battery of methods to survey the global features of the cells, from DNA, RNA and proteins to small molecules (Ideker *et al*., 2001; Kitano, 2002).

Transcriptome analysis, enabled by technology such as oligonucleotide microarray, is a simultaneous interrogation of gene expression by measuring the transcriptional activity on a global scale (Lockhart *et al*., 1996). Microarray, as well as the genome project, were the first forays of humanity into the realm of systems biology. Other than the fact that microarray analysis suffers from inherently noisy information (therefore requiring many replicates and often limited by cost and availability of materials), the other problem is the sheer volume of information obtained from this type of experiment (Claverie, 1999). Furthermore, the expression level change of a gene may be corollary to change in another gene and may not be the direct cause of the cellular phenotype. Additional information is required to place these genes in context.

Interactome analysis is a study of interactions between the biological molecules on a global scale (Ito *et al*., 2001). High-throughput mapping of protein interaction allows the global survey of protein interaction of organisms. The resulting maps of proteome-wide protein interactions are called protein networks.

Topological features of the protein networks have been demonstrated to reflect the functionality of the interacting genes. For example, essential genes in yeast tend to be well connected and globally centered in the protein network (Jeong *et al*., 2001; Wuchty and Almaas, 2005). Furthermore, globally centered interactions are more likely to be well conserved and serve as an evolutionary backbone for the network (Wuchty and Almaas, 2005).

Protein network analysis will place the genes identified in microarray experiments in a broader biological context. Since protein networks reflect the functional grouping of these interacting or coordinately induced/suppressed genes, the roles of the subsets of co-expressed genes may be resolved using the combined data. This may be done by evaluating the topological features of the sets of genes identified by microarray experiments.

Studies in cancer have validated the effectiveness of the microarray technique, allowing identification of tumor subclasses and marker genes for diagnosis and treatment of the disease (DeRisi *et al*., 1996; Sorlie *et al*., 2001). However, the genes identified have no further association with other genes that are co-regulated. Integration of protein network data may extend the reach of the established method of analysis by considering the genes in a broader context. Based on this notion, we seek to reveal the biological significance of differentially expressed genes in squamous cell lung cancer that is identified through our recent microarray gene expression profiling study by using interactome-transcriptome analysis. We find high centrality in these differentially induced genes, but not for the genes that are suppressed in cancer.

## 2 Materials and Methods

### 2.1 Sample preparation

Tissue biopsy samples were collected from five squamous cell lung cancer patients undergoing surgical removal of tumor. Total RNA was doubly extracted from these samples using TRIzol™ reagent (Invitrogen, Carlsbad, CA), following the manufacturer's instructions (Chomczynski and Sacchi, 1987).

### 2.2 Microarray analysis

The double-extracted total RNA was submitted to our Institute's core microarray facility. cRNA samples were prepared and hybridized to the array (Affymetrix® Hg-U133A™); its signals were then scanned using the protocols suggested by the manufacturer.

Bioconductor (Dudoit *et al*., 2003), a biological data analysis package based on R statistical programming language (Ihaka and Gentleman, 1996), was used for array data analysis and integration with other gene annotations. Robust microarray analysis (RMA) was used for normalization (Irizarry *et al*., 2003). RMA-derived expression values were used for the rest of the analysis (Galfalvy *et al*., 2003). Paired *t*-tests were used to distinguish the genes in which expression levels in the cancer cells differed from the paired normal lung tissue. Paired *t*-tests were possible because the control samples were taken from the normal lung tissues of the same individuals from whom the lung cancer samples were obtained.

### 2.3 Integration of array data to protein network

Online predicted human interaction database (OPHID, obtained on April 26, 2005) was used for the analysis of human protein interaction (Brown and Jurisica, 2005). Briefly, OPHID contains 16 034 known human protein interactions obtained from various public protein interaction databases, as well as 23 889 additional protein interactions that are predicted.

Genes in the array were matched to those in OPHID using gene symbols and protein sequences. In order to identify which genes in OPHID corresponded to which genes listed in the array data, we have used the following methods: (1) using the gene symbol that is directly indicated in the OPHID protein database and (2) using FASTA program to compare the peptide sequence of the OPHID protein to the peptide sequence of the array probe targets (Pearson and Lipman, 1988). As a result, 2137 genes on the microarray were matched to the protein network from OPHID.

### 2.4 Connectivity analysis of protein interaction map

Analysis was conducted on the connectivity of genes in the protein interaction map. For each connectivity *l*, genes in the protein network with exactly *l* links were selected (*n_l_*). From these genes, differentially expressed genes (DEGs) were counted $\left(n_{l}^{\prime }\right)$. The fraction $n_{l}^{\prime }/n_{l}$ was calculated (fraction genes, or FG) for each connectivity. Pearson's *r* was used to measure the correlation between the FG and the connectivity. In order to determine the significance of the correlation, the same number of genes that were differentially expressed was randomly chosen from the protein interaction map. Pearson's *r* of each set of randomly sampled genes was compared against the *r* obtained from DEGs to determine the likelihood that the correlation can occur by chance.

### 2.5 *k*-core analysis of protein interaction map

*k*-core analysis is an iterative process in which the nodes are removed from the graphs in order of least connected (Wuchty and Almaas, 2005). More specifically, for each iteration of *k*, given the network from the previous iteration, genes with less than *k* connections are removed from the graph. This will result in a series of subgraphs that gradually reveal the globally central region of the original network.

In order to measure the centrality of the selected set of genes, the excess retention (ER) of the differentially expressed genes was calculated for each *k*-core. ER is a measure of the degree to which proteins from a particular group are represented relative to the entire protein network. The detailed explanation of ER has been described elsewhere (Wuchty and Almaas, 2005).

## 3 Results

### 3.1 Expression profiles of lung cancer

To obtain a list of genes that are differentially expressed in cancer, tissue samples from five patients with squamous cell carcinoma (SCC) of the lung were collected. Additionally, an equal number of normal tissues surrounding the tumor were collected from the same patients for comparison. This control minimizes the effect of variation in gene expression between the individuals, yielding a more accurate characterization of the genes differentially expressed in the disease.

Following the normalization of the array data from these samples, genes that were consistently different from normal tissue (paired *t*-test, *P* < 0.05) were selected as DEG. Furthermore, only the genes that can be mapped to the existing protein network were chosen. As a result, 360 DEGs were chosen as genes that were upregulated in squamous carcinoma, whereas 270 DEGs were chosen as genes that were downregulated in squamous carcinoma (see online supplement for the list of genes). DEGs were subsequently mapped to protein interaction maps for further analysis.

### 3.2 Topological analysis of lung cancer genes

**Fig. 1:**
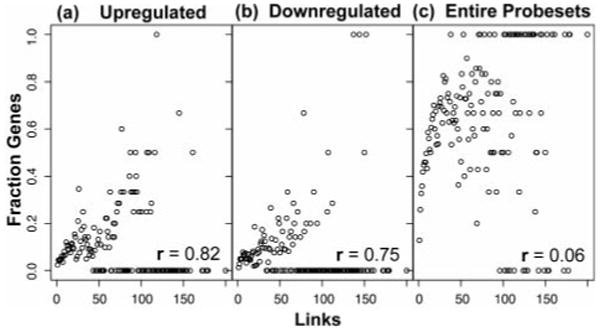
Correlation of connectivity (links) versus the fractions of select genes with exactly *l* links. The genes with exactly *l* links were chosen from the protein network, and then the fraction of selected genes from this subset was calculated. (**a**) Upregulated genes (*n* = 360) in SCC of lung have Pearson's *r*, which demonstrates high positive correlation (*r* = 0.82). (**b**) Downregulated genes (*n* = 270) in SCC have slightly less correlation (*r* = 0.75). (**c**) Microarray probesets that match the genes in the protein network (*n* = 2137) show no correlation to link number (*r* = 0.06). Thus, using the genes on the microarray does not contribute to bias in the number of links for genes differentially expressed in SCC. (FG values of 1 and 0 are not excluded for *r* values.)

In order to determine the topological features of the DEGs, the edge distribution for DEGs was compared to the rest of the graph (Section 2.4). We find that the genes that are upregulated in lung cancer have a positive correlation with the number of edges associated with them (Fig. 1a). This positive correlation indicates that lung cancer DEGs that are upregulated are highly connected. Down-regulated genes have a slightly lower, but positive correlation to connectivity (Fig. 1b).

Because only a subset of genes in the graph is present on the microarray that was used, bias of the selected DEGs is of concern. However, there is no correlation between the genes in the array to the connectivity in the protein network (Fig. 1c). Thus, the gene representation of the microarray itself poses no bias to the connectivity of the DEGs.

We then randomly selected the genes from the graph to see if we can get a high positive correlation. The same number of genes as DEGs was randomly sampled from genes that match the array. Then the correlation was calculated and compared the correlation to that of the DEGs. For the upregulated genes, average of 7.7% equaled or exceeded *r* of DEGs (*σ* = 1.8, *n* = 11). For the downregulated genes, average of 86.2% equaled or exceeded *r* of DEGs (*σ* = 0.8, *n* = 11). Since the only difference between the two analyses is the number of genes sampled (370 for upregulated genes and 270 for downregulated genes), the significance value is highly sensitive to the number of genes selected. Thus, one can conclude that, at a minimum, for the upregulated genes the positive correlation between the connectivity and the DEGs is not likely due to chance.

### 3.3 *k*-core analysis of protein network

**Fig. 2:**
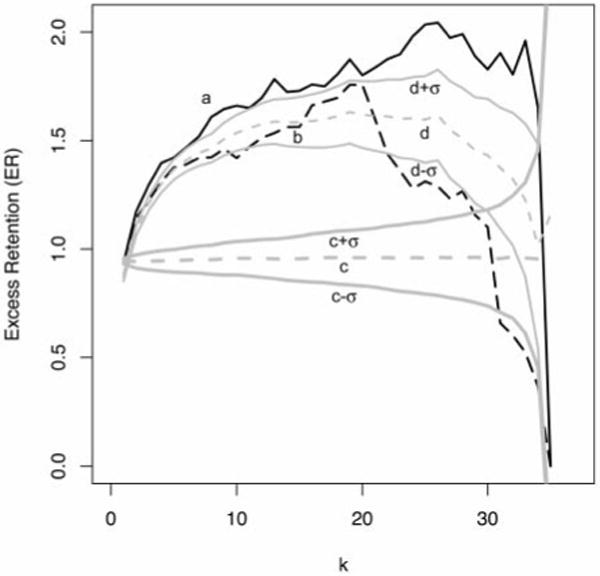
*k*-core analysis of differentially expressed genes in SCC of lung. *k* is the number of iterative decomposition of the graph (*k*-core subgraph), starting from the outermost edge. Higher *k* represents the central placement of the subset of genes in the original graph (*k*= 1). ER is the degree to which genes from a *k*-core is under- (ER < 1) or over-represented (ER > 1) relative to the original graph. a: ER of the upregulated genes (*n* = 360) in SCC; b: ER of the downregulated genes (*n* = 270) in SCC; c, c + *σ*, c − *σ*: mean ER of 1000 randomly selected genes from the entire network (*n* = 360), plus or minus the standard deviation (±*σ*); d, d + *σ*, d − *σ*: mean ER and ±*σ* for the 1000 randomly selected genes (*n* = 360) present in the microarray used in the experiment that matches the genes in the graph (*n* = 2137).

Using the *k*-core analysis method, we have measured how DEGs were close to the topological center of the protein network (Fig. 2). In this analysis, an increase in excess retention as value of *k* is increased indicates that the selected group of genes is located near the topological center of the protein network. Higher the *k* the ER is maintained, the closer the genes are to the center of the network. For the upregulated genes, the excess retention (ER) increases as *k* approaches 30, then drops precipitously around 35 (Fig. 2a). For the downregulated genes, ER increases as *k* approaches 20 and then descends beyond this point (Fig. 2b). The early peak of the downregulated genes relative to that of the upregulated genes indicates that the upregulated genes are more centrally located in the protein network than the downregulated genes.

In order to test the significance of this finding, ER has been calculated for the same number of nodes that has been randomly selected from the graph. When genes were chosen from the entire graph, ER remained near unity, on average (Fig. 2c). However, when genes that correspond to the microarray probesets were chosen, ER did increase on average (Fig. 2d) up to *k* = 20. A distinct difference in the ER for each *k*-core can be seen between cancer gene and random control (average of 7 out of 1000 randomly chosen genes out of array probes are above the ER for upregulated genes where 1 < *k* < 35). Moreover, it is apparent that downregulated genes do not show a significant difference in ER against the genes that were randomly chosen from the arrays. From these results, we can conclude that the upregulated genes are centrally located in the protein network, but the same conclusion cannot be made for the downregulated genes.

## 4 Discussion

Transcriptional profiling of cancer using microarray has revolutionized the field by allowing researchers to discover tumor subclasses and target genes for diagnosis and therapy. Protein interaction mapping using high-throughput yeast-two-hybrid (HT-Y2H) methods has been hailed as the harbinger for the systematic approach to functional genomics, allowing individual genes to be placed in a global context of cellular functions (Mendelsohn and Brent, 1999). This leads to the question how are genes that are differentially regulated in cancer placed in the global context of the protein interaction map? In this study, we exploit the most recent advances in interactome analysis to answer this question.

Much of the biological diversity of tumors is the result of variation in the transcriptional programs (Perou *et al*., 2000). Microar-ray analysis of cancer cells has allowed the identification of specific genes or proteins that could serve as molecular targets for improved diagnosis and therapy. The subsequent interpretation of the genes identified by the microarray analysis was analyzed in a context where a single gene was accounted for the individual phenotype. However, this ignores the fact that proteins rarely act in isolation from one another, and their specific functions are determined by association with other proteins.

A rational starting point for taking the proteins into proper context is to use the protein network or interactome. Interactome analysis has made a remarkable progress in the past decade, mainly due to the development of high-throughput screening methods such as HT-Y2H. To date, interactomes of three eukaryotes have been mapped by HT-Y2H (Giot *et al*., 2003; Li *et al*., 2004; Uetz *et al*., 2000). Due to high cost and labor, human HT-Y2H maps may not be available for some time. Predicted mapping of human protein interaction can serve as a surrogate map, years before the complete human protein network based on direct experimental evidence becomes available.

Hypothetical human protein interaction maps are relatively new (Brown and Jurisica, 2005; Lehner and Fraser, 2004). The prediction of protein interaction is based on sequence-based searches for conserved protein interactions, also known as interologs (Matthews *et al*., 2001). Because these human interaction maps are hypothetical, it is likely to have many false positives as well as missing protein interactions. There is a claim that approximately half of the predicted interactions using interologs between microorganisms can be experimentally validated (Sharan *et al*., 2005). While the lack of accuracy in the interologs approach may instill little confidence in the predicted map, it is easy to envision its role in the validation of the HT-Y2H global protein network mapping.

The topological analyses using a hypothetical human protein interaction map suggest that the genes upregulated in the squamous lung carcinomas are rich in global hubs. Global hubs are well-connected nodes that are also located near the center of the protein network. Evidence is lacking to make the same claim to the down-regulated genes. Centrality of the genes is associated with the essential functions of the genes in the yeast (Jeong *et al*., 2001). It has been shown that the essential genes, those that are lethal when mutated, tend to be well connected. Another study shows that the yeast genes that are not essential but provide a vital function in toxin metabolism also have high number of edges associated with the nodes, albeit less well-connected than that of the essential genes (Said *et al*., 2004). *k*-core analysis has been performed on the yeast essential genes and were shown to be global hubs, whereas non-essential genes were not (Wuchty and Almaas, 2005). The study also indicates that these global hubs are conserved throughout different species.

One can surmise, from the centrality of the cancer-associated genes, the evolutionary constraints to the genes expressed in cancer cells. Whatever the mechanism, there must be a core set of genes that needs to be maintained throughout the course of somatic evolution in the tumor microenvironment. These genes are the ‘essential genes’ for the cancer and share the topological characteristics that the essential genes do in yeast. This may be manifested by the upregulated genes in our cancer samples that tend to be, but not restricted to, the global hubs. Downregulated genes tend to be less constrained in this way, either because the genes are suppressed by the upregulated genes (secondary effect of the core cancerous gene expression) or because the suppressed genes are overwhelmed by circumvention from the alternate or compensatory pathway that induces the upregulation of the cancerous genes.

Our analysis concludes that squamous cell lung cancer genes share similar topological features for essential proteins. This finding fulfills the somatic evolution model of cancer. Cancerous cells undergo frequent mutation, division and selection, ultimately leading to the fittest ‘disregulated’ phenotype. Given this, it is reasonable to assume that the genes, which are differentially expressed in contrast to the surrounding normal tissue, are essential for survival and proliferation. Thus, given that the centrality of the genes in a protein network is characteristic of the essential genes in yeast, it follows that the genes essential for cancer cells would also share the topological characteristics of these essential genes.

This is the first time the predicted human protein interaction map has been used for the analysis of cancer. While microarray analysis has been used extensively for the identification of marker genes for various types of cancer, our approach may facilitate the development of drugs that target the genes that may be directly responsible for the disease.

## Supplementary Material

1
