# Supplementary material for: Interactome-transcriptome analysis reveals the high centrality of genes differentially expressed in lung cancer tissues
Source: Bioinformatics. Author manuscript; Available in PMC 2015 Nov 3. (PMC4631381; doi:10.1093/bioinformatics/bti688)
Supplement: 1 [file NIHMS667662-supplement-1.pdf]

Supplementary Information

Additional Tables:

File **expval.table.txt** contains the list of genes that has been determined to be differentially regulated. Since HgU133A microarray often includes multiple probesets of a same gene, these were also included. Full dataset has been submitted to NCBI's GEO database under accession number GSE3268.

Row header is indicated in the first line. First column, **hgu133a** are the probeset identifiers used in the microarray. **X__nml** and **X__sqm** are the calculated expression values for normal and tumor sample of patients (respectively), where underscores (__) represent each individual's identification number. **Symbol** column contains the official symbol for the genes. **meanD** column contains the mean difference in gene expression between the tumor and cancer sample. **p-val column** contains the p-value of paired t-test for the expression values of the gene. **Updn** contains the final verdict for the gene, whether the tumor sample over- or under-expresses a gene in tumor. **maxk** column contains the maximum *k* value for the each gene using the *k*-core analysis.

File **fig1tbl.txt** contains the table of values plotted on Figure 1 in the paper.

Section 2.3: Matching of Affymetrix Oligo Probeset Targets with OPHID proteins

The following processes were used in order to compare the transcriptome data, obtained by Affymetrix oligonucleotide microarray, with the genes are present in the protein network represented by OPHID. File **ophid1114237070973.txt** is the original file downloaded from OPHID database, which was used for the analysis in this study.

First, official gene symbol (determined by HUGO Gene Nomenclature Committee, used by both OPHID and Affymetrix) were used to match the probesets in Affymetrix microarray and OPHID. OPHID database includes Swissprot ID and gene symbol. For each Swissprot ID, the matching symbol was extracted from the file.

Affymetrix probe dataset is downloaded as a source from Bioconductor web site. The Bioconductor metadata was built from data obtained on LocusLink, built on March 3, 2004. Simple table matching was performed using R code. Some of the genes were not matched by symbols, because some of the genes were not given standard gene symbols. For these cases, FASTA sequence matching program was used. Protein sequence for Affymetrix HGU133A target genes were obtained from EnsMART at ENSEMBL. OPHID contains the protein sequence used for their protein network generation. Using these two tables, we have performed pair-wise sequence alignment to find the best matching case. We have used the default setting for FASTA program, and discarded where the p value of the match exceeded 0.01. File **swiss2sym.txt** includes the completed conversion table.

Section 3.3: *k*-core Analysis of Protein Network

To implement the *k*-core analysis the following steps were used.

1. Sort nodes according to their present degree (connectedness)
    a. Determine the existing nodes from the paired list
    b. Determine the degree of nodes
2. Remove the nodes with degree lower than *k*
    a. Create a selection list for edges with more or equal to *k* degrees
    b. Select according to list.

The following is the **R code** used for the analysis of **the.graph**, which is a binary list (2-by-*n* matrix) of genes, which is derived from OPHID.

```
nodedeg <- function(the.graph, counter = F){
        x <- unique(as.character(the.graph))
        y <- as.numeric(x)
        y[] = c(0)
        names(y) = x
        node.deg <- y
        for(i in x){
          if(counter){
          cat(i,"\n")}
          node.deg[i] = sum(the.graph[,1] == i) + sum(the.graph[,2] == i)
        }
        node.deg
}

k.core <- function(the.graph, k){
        nd <- nodedeg(the.graph)
        qnd <- names(nd[nd >= k])
        the.graph[(the.graph[,1] %in% qnd) & (the.graph[,2] %in% qnd),]
}
```

Iteration of this will be...

```
k.core.result <- c()
k.core.result[[1]] = the.graph
for(i in 2:9)
{
  cat(i,"core\n")
  the.graph <- k.core(the.graph, i)
  k.core.result[[i]] = the.graph
}
```

Thus, **k.core.result** will be a list of graphs with *m* elements, where the iterative pruning occurs incrementally.

Here is the code for the calculation of excess retention (*ER*). The description of the calculation is detailed in section 2.3 of this paper. **A** is the list of genes in the graph.

```
#count # nodes in graph
z <- unique(as.character(the.graph))
N <-  length(z)
Na <- sum(z %in% A)
Ea <- Na / N

ERak.list <- c()
for(i in 1:9)
{
  kc <- k.core.result[[i]]
  z <-  unique(as.character(kc))
  Nk <- length(z)
  nak <- sum(z %in% A)
  eak <- nak/Nk
  ERak <- eak/Ea
  ERak.list[i] = ERak
}
```

Thus, **ERak.list** contains the list of *ER* values for each *k*-core for the given list of genes.
